# The effectiveness of installing trackside fencing in preventing railway suicides: a pre-post study design in Victoria, Australia

**DOI:** 10.1136/ip-2023-044897

**Published:** 2023-08-14

**Authors:** Angela Clapperton, Jeremy Dwyer, Matthew Spittal, Jane Pirkis

**Affiliations:** 1 Centre for Mental Health, Melbourne School of Population and Global Health, The University of Melbourne, Melbourne, Victoria, Australia; 2 Coroners Prevention Unit, Coroners Court of Victoria, Melbourne, Victoria, Australia

**Keywords:** suicide/self?harm, environmental modification, engineering

**WHAT IS ALREADY KNOWN ON THIS TOPIC**

- Restricting access to means has been shown to be an effective suicide prevention measure in public places in general, and in the railway environment specifically.
- Some examples of restricting access to means that have been effective in the railway environment include the installation of platform screen doors and the removal of level crossings.

**WHAT THIS STUDY ADDS**

- Our study adds to knowledge about restricting access to means in the railway environment by examining the effectiveness of the installation of trackside fencing, and in particular, by examining whether differences in effectiveness exist depending on the length of the fencing installed.
- We identified a 57% reduction in suicides if the fencing was at least 100 metres long.

**HOW THIS STUDY MIGHT AFFECT RESEARCH, PRACTICE OR POLICY**

- This finding has implications for designing barrier interventions like trackside fencing and selecting fencing locations to prevent railway suicide.
- Our study suggest that authorities who are responsible for installing fencing on railway networks may wish to prioritise sites where longer runs of fencing can be installed.

## Introduction

Railway suicide, while relatively rare—estimated to account for 1–12% of suicides internationally^1 2^ —carries extensive related psychological costs for family and friends of the deceased as well as for train drivers and witnesses, and the financial implications are substantial.^3^ More than 40% of Australian suicides by jumping or lying before a moving object (mostly trains) occur in the state of Victoria^4^ where most of the railway network is unfenced.

Many different measures have been implemented in an effort to prevent railway suicide, including those that restrict access to means (eg, physical barriers such as platform screen doors^5–8^), those that encourage help seeking (eg, signs indicating sources of help^9^), those that increase the likelihood of intervention by a third party (eg, training of railway staff or bystanders^10 11^) and those that encourage responsible media reporting of railway suicides (eg, media guidelines^12^). Our study is concerned with the first of these types of interventions—restricting access to means. Restricting access to means in the railway environment can occur in a number of ways. For example, the track can be restricted by the geography of the surrounding environment (eg, lakes, rivers, inaccessible land) or through locating the railway track in tunnels. Examples of restricting access to means working effectively in the railway environment to reduce suicide, include the installation of platform screen doors in stations,^5–8^ and removal of level crossing sites.^4^ Installing new fencing, or upgrading existing fencing, on the railway network is another way to potentially reduce access to means. Mid-track fencing, which is fencing placed in between high-speed and commuter train tracks to restrict access to the high-speed train tracks, has reduced the number of railway suicides by 62.5% at a station where it has been installed in Sweden.^13^

In some locations across the world a high proportion of railway suicides occur at stations.^14^ However, in other locations such as the USA,^15^ Germany^16^ and in Victoria, Australia^17^ the majority of railway suicides take place on open tracks. In Victoria the high proportion occurring on open tracks is presumably a result of much of the railway network being unfenced, and is therefore easily accessible by someone considering this method of suicide.^18^ In recent years, small amounts of standard fencing (not mid-track fencing) have progressively been installed on the railway network in metropolitan Victoria. This fencing has been installed to prevent intruders accessing the track at known problem locations on the railway network. In some locations the fencing has been installed on both sides of the track while at others the fencing has been installed on only one side of the track. To examine whether this was an effective railway suicide prevention measure, we used data from the Victorian Suicide Register (VSR) to test whether the incidence of railway suicides occurring near to sites where fencing was installed changed after the installation of the fencing.

## Methods

### Study design

We used a pre-post design to test whether the installation of fencing was associated with a decrease in railway suicides within a 500 metre radius and 1000 metre radius of the mid-point of the fencing sites. We compared the incidence of suicide before and after the period that the fencing was installed (i.e., in the pre- and post- intervention periods) by calculating rate ratios. The length of the fencing at sites ranged from 14 metres to 1370 metres so we also examined whether there was a different effect when fences shorter than 100 metres were compared with fences that were 100 metres or longer.

### Data sources

We obtained information from Metro Trains Melbourne which allowed us to identify 36 locations where fencing was installed between January 2017 and December 2020, and we extracted suicide data from the VSR for the period January 2008 to June 2021.

We used the information contained in the VSR, which includes the longitude and latitude of all incidents, to identify all railway suicides occurring within a 500 metre radius and a 1000 metre radius of the mid-point of fencing sites over the period January 2008 to June 2021. We did not know the exact dates of fencing installation at each site only the calendar year in which fencing was installed, so the pre-period we used for analysis included full calendar years prior to any work beginning and the post-period included full calendar years covering the period when the work had concluded.

### Analysis

For the fencing sites we compared the rate of railway suicide in the pre- and post-intervention periods (ie, before and after the installation of the fencing), calculating rate ratios. We did this for railway suicides within a 500 metre radius of the fencing site and 1000 metre radius of the fencing site. We also conducted the analysis separately for sites with fencing shorter than 100 metres and for sites with fencing that was 100 metres or longer. We calculated the rates as: (1) the number of suicides divided by the total number of months in the pre-intervention period and (2) the number of suicides divided by the total number of months in the post-intervention period. The rate ratio was then the ratio of these two incidence rates. Confidence intervals were calculated using the exact method.

### Ethics

The study was reviewed and approved by the University of Melbourne’s Human Research Ethics Committee (Reference Number: 2021-22015-21133–3).

## Results

Over the period January 2008 to June 2021, we identified 103 railway suicides that occurred within a 1000 metre radius of the mid-point of a site where fencing was installed (table 1).

**Table 1:** Number of railway suicides occurring within a 500 metre and 1000 metre radius of fencing sites and rate ratios (RR) comparing the pre- and post-intervention periods, Victoria, January 2008 to June 2021

	Pre-period			Post-period			
	Number of suicides	Number of months	Suicide rate (per month)	Number of suicides	Number of months	Suicide rate (per month)	RR (95% CI)
500 metre radius of site							
All sites (36 sites; 7995 m)	43	4584	0.009	*	816	0.005	0.52 (0.14 to 1.44)
Sites with fences <100 m (nine sites; 399 m)	9	1164	0.008	*	186	0.005	0.70 (0.02 to 5.02)
Sites with fences ≥100 m (27 sites; 7596 m)	34	3420	0.010	*	630	0.005	0.48 (0.09 to 1.52)
1000 metre radius of site							
All sites (36 sites; 7995 m)	93	4584	0.020	10	816	0.012	0.60 (0.28 to 1.16)
Sites with fences <100 m (nine sites; 399 m))	18	1164	0.015	*	186	0.022	1.39 (0.34 to 4.22)
Sites with fences ≥100 m (27 sites; 7596 m)	75	3420	0.022	*	630	0.010	0.43 (0.15 to 0.99) †

*Cell suppressed due to small cell count.

†Significant decrease.

When we examined all sites (regardless of the length of fencing installed), we found no evidence that the number of railway suicides within a 500 metre radius or a 1000 metre radius of a fencing site decreased after the installation of fencing (500 m RR: 0.52; CI 95% 0.14–1.44; 1000 m RR: 0.60; CI 95% 0.28–1.16) (table 1).

However, there was evidence that when the fencing was 100 metres or longer the incidence of railway suicide decreased by 57% within a 1000 metre radius of fencing sites (RR: 0.43; CI 95% 0.15–0.99).

## Discussion

Through analysis of suicide surveillance data in Victoria we found no evidence of an overall reduction in the rate of railway suicides within a 500 metre radius and 1000 metre radius of fencing sites when all sites and all lengths of fencing were considered. It is perhaps not unexpected that a reduction in suicides did not occur, given at many sites the fencing was quite short and therefore did not fully restrict access to the track over the distances that we examined (i.e., 500 metre radius and 1000 metre radius of the mid-point of the fencing).

Importantly, when we examined the effectiveness of fencing separately for fencing shorter than 100 metres and for fencing that was 100 metres or longer, we were able to identify a 57% decrease in the rate of railway suicides within a 1000 metre radius of sites where the fencing was more than 100 metres in length. This finding is relatively consistent with other suicide prevention research that has demonstrated superior results of interventions that completely, rather than partially, restrict access to means at a site. For example, using full-height screen doors on railway station platforms^5 7 8^ and barriers on bridges that secure the whole bridge^19^ are more effective than interventions that only partially restrict access.

The main strength of our study was the suicide data that we used. We used real-time suicide register data which allowed us to examine the effect of fencing that had only been installed recently. In addition, the data included accurate, manually assigned, geocoded incident location data. Manual coding is important because relying on data based on auto-geocoding processes can be problematic for incidents occurring in public places as they can be erroneously geocoded to centralised fall-back locations.^20^

Our study also had some limitations. We did not examine potential substitution effects, where railway suicides may have increased at other sites where fencing was not installed. This is an important area for future study given that the Swedish study mentioned above showed that following a reduction in suicides at a site where mid-fencing was installed there was some evidence of an increase in suicides at nearby stations without mid-track fences.^13^ Our lack of control sites for the study means our analysis did not account for other potentially relevant changes in the railway environment that have occurred during the period of study (e.g., level crossing removals, which we showed decreased railway suicides near to removal sites^4^). However, we were able to determine that four of the fencing sites included in our study were within 1000 metres of level crossing sites that were removed at some point during the study period. We re-ran our analysis excluding the four sites and found that the results were very similar although the magnitude of some results changed slightly. Finally, there was a low number of suicides where fencing was shorter than 100 metres and this means these results may suffer strength and magnitude errors.

Overall, our study findings suggest that the length of fencing on the railway network may be an important determinant of its effectiveness in reducing railway suicide; we found a significant suicide prevention effect of fencing installed to prevent intruders accessing the track at known problem locations on the railway network, only when the fencing was greater than 100 metres in length. Authorities who are responsible for installing fencing on railway networks may wish to prioritise sites where longer runs of fencing can be installed.
